# A New Method of Treating Subcutaneous Nævi

**Published:** 1881-11

**Authors:** Carey Coombs

**Affiliations:** London


### Selections.

### A New Method of Treating Subcutaneous Nævi.

By Carey Coombs, M. D., London.

About a year ago a child, aged nine months, was brought to me with a nævus about three-quarters of an inch in diameter, filling up the fossa on the left side of the nose. The swelling was entirely subcutaneous, and it was evident that none of the applications which cure the superficial form of the disease would be of any use. The gold needle usually employed in such cases was connected with a battery and introduced into the tumor, but the restlessness of the child (his eye being endangered) made me abandon it. Two lengths of No. 24 silver wire were then passed through the middle of the swelling, parallel to each other, and about a quarter of an inch apart. The zinc and carbon of a Bunsen cell (quart size) were then connected with the ends of each wire separately. The result was great heat in the wire during the short period, one or two seconds, of connection. The ends of the wires were then tightly twisted together, protected by being covered with lint and plaster, and left for the next application, which took place a week later. The current was applied three times altogether. The wires were removed after the third galvanization, and no further treatment was needed. The nævus is now scarcely perceptible.

This mode of using the galvanic current in the deeper nævi appears to me to be recommended by its simplicity and freedom from danger. There is less pain than is caused by the usual introduction of needles at each operation, and a single cell (bichromate, Grove's, or Bunsen's) is sufficient, the only resistance being the fine silver wire.—*London Lancet.*

